# Sarcopenia Risk Identified by Strength, Assistance With Walking, Rising From a Chair, Climbing Stairs, and Falls (SARC-F) in Patients With Cirrhosis: A Real-World Cross-Sectional Study

**DOI:** 10.7759/cureus.104996

**Published:** 2026-03-10

**Authors:** Noor Albusta, Ali Yusuf, Ahmed Ali

**Affiliations:** 1 Internal Medicine, Beth Israel Lahey Health, Burlington, USA; 2 Medicine, Bahrain Government Hospitals, Manama, BHR

**Keywords:** chronic liver disease, cirrhosis, sarc-f, sarcopenia, screening

## Abstract

Introduction: Sarcopenia is highly prevalent in patients with liver cirrhosis and is associated with increased morbidity, hospitalization, and mortality. However, routine screening is not consistently performed in clinical practice. The strength, assistance with walking, rising from a chair, climbing stairs, and falls (SARC-F) questionnaire is a simple tool that may facilitate early identification of sarcopenia risk.

Methods: This cross-sectional study included adult patients with cirrhosis attending a tertiary government hospital in Bahrain between January 1, 2024, and December 31, 2025. Sarcopenia risk was assessed using the SARC-F questionnaire, with a score ≥4 indicating high risk. Clinical and laboratory data were collected. Multivariable logistic regression was used to identify independent predictors of sarcopenia risk.

Results: A total of 362 patients were included (mean age 56.8 ± 11.9 years; 221 (61.0%) males). High sarcopenia risk (SARC-F ≥4) was identified in 129 patients (35.6%). Patients with high risk were older (60.2 vs 54.9 years, p < 0.001), had higher model for end-stage liver disease (MELD) scores (14.6 vs 11.2, p < 0.001), and more frequent ascites (75 (58.1%) vs 80 (34.5%), p < 0.001). They also had higher rates of hospitalization (55 (42.6%) vs 54 (23.3%), p < 0.001). On multivariable analysis, age (adjusted odds ratio (aOR): 1.05; 95% CI: 1.02-1.07), MELD score (aOR: 1.09; 95% CI: 1.04-1.14), and ascites (aOR: 1.87; 95% CI: 1.18-2.95) were independently associated with sarcopenia risk.

Conclusions: Sarcopenia risk appears to be common among patients with cirrhosis and is associated with markers of disease severity. The SARC-F questionnaire may serve as a simple screening tool to identify patients at higher risk in routine clinical practice.

## Introduction

Liver cirrhosis represents the final stage of chronic liver disease and is associated with significant morbidity and mortality worldwide [1]. In addition to hepatic complications, patients with cirrhosis frequently develop extrahepatic manifestations, including sarcopenia, which is increasingly recognized as a key determinant of outcomes in this population [1].

Sarcopenia, defined as the loss of skeletal muscle mass and function, affects up to 30%-70% of patients with cirrhosis, depending on the diagnostic criteria used [1,2]. A recent meta-analysis of 55 studies involving over 13,000 patients reported an overall prevalence of 40.1% (95% CI: 35.4%-44.9%), with higher rates observed in males, those with decompensated cirrhosis, and patients with alcohol-associated liver disease [2]. Sarcopenia is associated with higher rates of hepatic decompensation, infections, hospitalization, and mortality, independent of liver disease severity scores such as the model for end-stage liver disease (MELD) [1,3]. A meta-analysis of 22 studies demonstrated that sarcopenia was independently associated with an approximately two-fold increased risk of mortality in patients with cirrhosis (adjusted hazard ratio: 2.30; 95% CI: 2.01-2.63) [2].

Despite its clinical significance, sarcopenia remains underrecognized in routine practice [1]. Traditional diagnostic methods, including computed tomography (CT)-based muscle mass assessment at the level of the third lumbar vertebra or dual-energy X-ray absorptiometry (DEXA), are considered reference standards for evaluating muscle mass and quality [1,4]. However, these modalities require specialized equipment, trained personnel, and, in the case of CT, radiation exposure, making them unsuitable for routine outpatient screening [1]. This has led to increased interest in simple bedside tools that can identify patients at risk.

The SARC-F questionnaire is a validated, patient-reported screening tool that assesses five domains: strength, assistance with walking, rising from a chair, climbing stairs, and history of falls [2]. Each component is scored from 0 to 2, yielding a total score ranging from 0 to 10, with a score of 4 or greater indicating high sarcopenia risk. It has been recommended as a case-finding instrument for sarcopenia in various populations, including those with chronic diseases, due to its low sensitivity but high specificity [2,5]. In patients with chronic liver disease, SARC-F has demonstrated utility in identifying those at risk of sarcopenia and adverse outcomes, with the frequency of high SARC-F scores increasing significantly with progression of liver disease [2,6].

Data on the prevalence of sarcopenia risk and its clinical correlates in Middle Eastern populations with cirrhosis remain limited. Understanding the burden of sarcopenia in this region is particularly important given the rising prevalence of metabolic dysfunction-associated steatotic liver disease (MASLD) and the demographic transition toward an aging population with increasing rates of chronic liver disease [1]. Furthermore, identifying high-risk subgroups may inform targeted interventions aimed at preventing or reversing muscle loss in this vulnerable population.

This study aims to evaluate sarcopenia risk using the SARC-F questionnaire in patients with cirrhosis attending a tertiary government hospital in Bahrain and to assess its association with clinical characteristics and healthcare utilization. Specifically, the primary objectives were (1) to estimate the prevalence of high sarcopenia risk (SARC-F ≥4) and (2) to identify independent clinical predictors associated with elevated SARC-F scores in this population. Given the cross-sectional design, this analysis is intended to be hypothesis-generating and to evaluate associations rather than causal relationships.

## Materials and methods

Study design and setting

This cross-sectional study was conducted at a tertiary care government hospital in Bahrain between January 1, 2024, and December 31, 2025. The study was conducted and reported in accordance with the Strengthening the Reporting of Observational Studies in Epidemiology (STROBE) guidelines [7]. The study was approved by the institutional research ethics committee, and written informed consent was obtained from all participants prior to enrollment.

Study population

Inclusion Criteria

Adult patients aged 18 years or older with a diagnosis of cirrhosis were eligible for inclusion. Cirrhosis was diagnosed based on clinical findings (stigmata of chronic liver disease, portal hypertension), radiological evidence (nodular liver contour, splenomegaly, or portosystemic collaterals on ultrasound or CT), liver stiffness measurement exceeding 15 kPa on transient elastography, or histological confirmation when available. 

Exclusion Criteria

Patients were excluded if they had acute liver failure, hepatocellular carcinoma beyond Milan criteria, severe cognitive impairment preventing questionnaire completion, or inability to provide informed consent.

Data collection

Clinical and demographic data were collected from medical records at the time of enrollment. Variables included age, sex, etiology of cirrhosis, and comorbidities, including diabetes mellitus and hypertension. A history of hepatic decompensation was recorded, including prior episodes of ascites, variceal bleeding, and hepatic encephalopathy. Laboratory parameters obtained within 30 days of enrollment included total bilirubin, international normalized ratio (INR), serum creatinine, and serum albumin. The MELD score was calculated using the standard formula incorporating bilirubin, international normalized ratio (INR), and creatinine [8]: \[\text{MELD} = 9.57 \cdot \ln(\text{creatinine}) + 3.78 \cdot \ln(\text{bilirubin}) + 11.2 \cdot \ln(\text{INR}) + 6.43\]

Healthcare utilization data, including the number of hospital admissions in the preceding 12 months, were extracted from electronic medical records.

Assessment of Sarcopenia Risk

Sarcopenia risk was assessed using the SARC-F questionnaire, which was administered by trained research staff during outpatient clinic visits. The questionnaire evaluates five functional domains: strength (difficulty lifting or carrying 10 pounds), assistance with walking (difficulty walking across a room), rising from a chair (difficulty transferring from a chair or bed), climbing stairs (difficulty climbing a flight of 10 stairs), and falls (number of falls in the past year) [9]. Each item is scored from 0 to 2, with higher scores indicating greater difficulty. The total score ranges from 0 to 10, and a score of 4 or greater was used to define high sarcopenia risk, consistent with established cutoffs [9].

Outcomes

The primary outcome was the prevalence of high sarcopenia risk, defined as a SARC-F score of 4 or greater. Secondary outcomes included the association between sarcopenia risk and liver disease severity as measured by MELD score, the presence of hepatic decompensation (ascites and encephalopathy), and healthcare utilization defined as one or more hospital admissions in the preceding 12 months.

Statistical analysis

Continuous variables were assessed for normality using the Shapiro-Wilk test and are presented as mean ± standard deviation for normally distributed data or median with interquartile range for skewed distributions. Categorical variables are presented as frequencies and percentages. Comparisons between patients with high sarcopenia risk (SARC-F ≥4) and low sarcopenia risk (SARC-F <4) were performed using the independent samples t-test or Mann-Whitney U test for continuous variables and the chi-square test or Fisher's exact test for categorical variables, as appropriate. Multivariable logistic regression was performed to identify independent predictors of high sarcopenia risk.

Variables with a p-value less than 0.10 in univariate analysis were entered into the multivariable model. In addition, clinically relevant covariates (including sex and diabetes mellitus) were considered during model construction to minimize omission of important confounders, even if they did not meet the univariate statistical threshold. The final multivariable model included five covariates with 129 outcome events, corresponding to approximately 25 events per variable, exceeding commonly recommended thresholds to reduce the risk of model overfitting. Multicollinearity among variables was assessed using variance inflation factors (VIF), and no significant collinearity was identified. Results are reported as adjusted odds ratios (aOR) with 95% CIs. Model fit was assessed using the Hosmer-Lemeshow goodness-of-fit test. All statistical analyses were performed using IBM SPSS Statistics for Windows, Version 28 (Released 2021; IBM Corp., Armonk, New York, United States), and a two-sided p-value of less than 0.05 was considered statistically significant. 

## Results

Baseline characteristics

**Figure 1:**
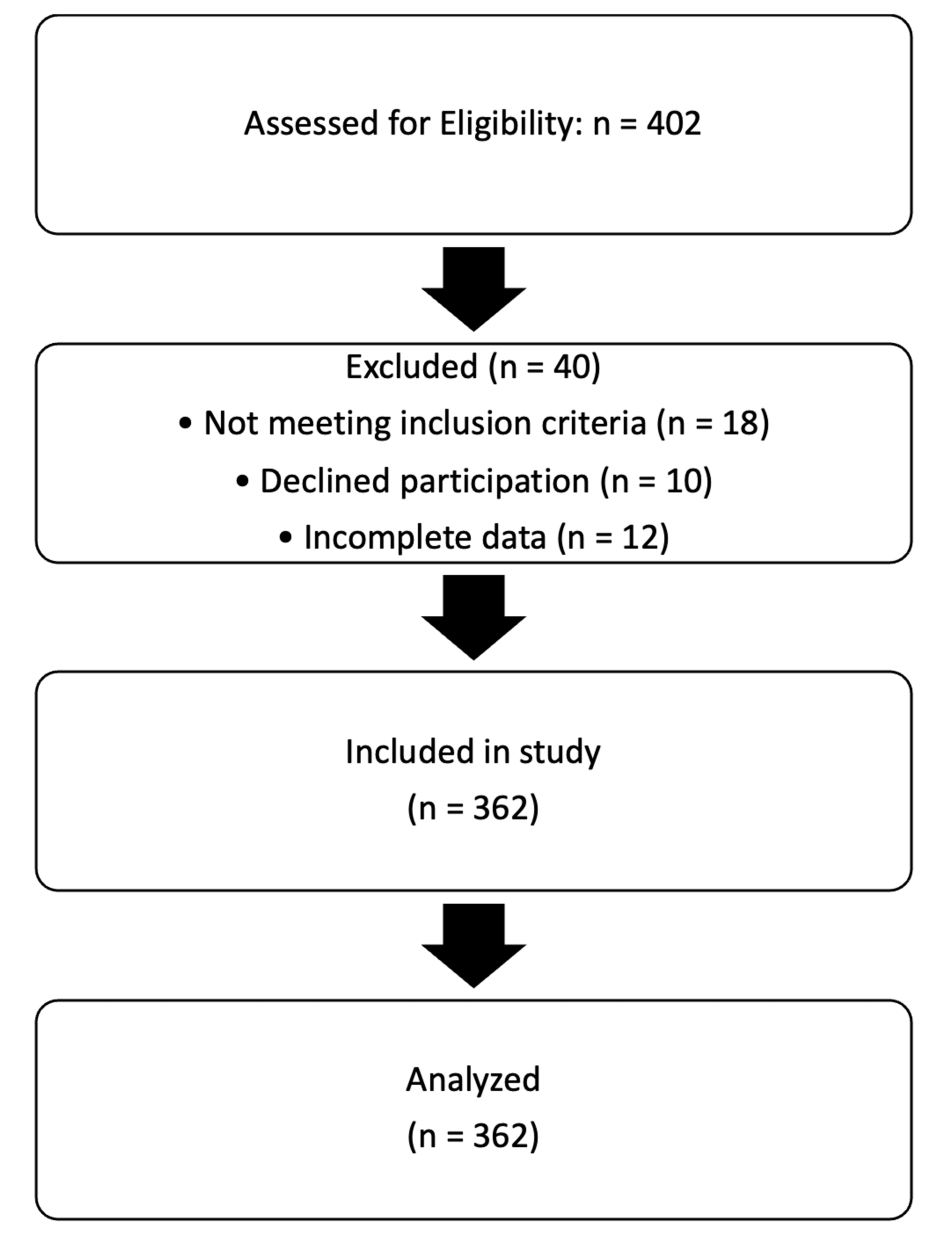
STROBE flow diagram of patient selection STROBE: Strengthening the Reporting of Observational Studies in Epidemiology

A total of 402 patients were screened for eligibility. Of these, 18 did not meet the inclusion criteria, 10 declined participation, and 12 were excluded due to incomplete data. Among the excluded patients with incomplete data, eight had missing SARC-F questionnaire responses, and four had missing laboratory values required for analysis. The excluded patients did not differ significantly from those included in the final cohort with respect to age (mean: 57.3 vs 56.8 years; p = 0.82), sex distribution (58.3% vs 61.0% males; p = 0.85), or MELD score (mean: 12.8 vs 12.4; p = 0.76). Ultimately, a total of 362 patients with cirrhosis were included in the final analysis (Figure 1). Of the 362 patients included, cirrhosis was diagnosed by clinical and radiological findings in 198 patients (54.7%), transient elastography in 142 patients (39.2%), and histological confirmation in 22 patients (6.1%). The mean age was 56.8 ± 11.9 years, and 221 patients (61.0%) were males. The most common etiology of cirrhosis was MASLD, accounting for 150 patients (41.4%), followed by alcohol-related liver disease in 99 patients (27.3%) and viral hepatitis in 76 patients (21.0%). The remaining 37 patients (10.2%) had cirrhosis attributed to autoimmune hepatitis, primary biliary cholangitis, or cryptogenic causes. The mean MELD score was 12.4 ± 4.9. Diabetes mellitus was present in 178 patients (49.2%), and hypertension was present in 201 patients (55.5%). Prior hepatic decompensation was common, with ascites present in 155 patients (42.8%), variceal bleeding in 63 patients (17.4%), and hepatic encephalopathy in 67 patients (18.5%). Patients may have more than one comorbidity or hepatic decompensation event; therefore, percentages are not mutually exclusive and may exceed 100% (Table 1).

**Table 1:** Baseline characteristics and clinical outcomes stratified by sarcopenia risk (SARC-F score) SARC-F: strength, assistance with walking, rising from a chair, climbing stairs, and falls; MASLD: metabolic dysfunction-associated steatotic liver disease

Characteristic	Overall (N = 362)	Low-risk SARC-F < 4 (n = 233)	High-risk SARC-F ≥4 (n = 129)	p-value
Demographics				
Age, years (mean ± SD)	56.8 ± 11.9	54.9 ± 12.1	60.2 ± 11.3	0.001
Male sex, n (%)	221 (61.0)	144 (61.8)	77 (59.7)	0.68
Etiology of cirrhosis, n (%)				
MASLD	150 (41.4)	88 (37.8)	62 (48.1)	0.06
Alcohol-related	99 (27.3)	68 (29.2)	31 (24.0)	0.29
Viral hepatitis	76 (21.0)	50 (21.5)	26 (20.2)	0.78
Other	37 (10.2)	27 (11.6)	10 (7.8)	0.27
Comorbidities, n (%)				
Diabetes mellitus	178 (49.2)	109 (46.8)	69 (53.5)	0.21
Hypertension	201 (55.5)	124 (53.2)	77 (59.7)	0.24
Liver disease severity				
MELD score (mean ± SD)	12.4 ± 4.9	11.2 ± 4.3	14.6 ± 5.2	0.001
Hepatic decompensation, n (%)				
Ascites	155 (42.8)	80 (34.5)	75 (58.1)	0.001
Variceal bleeding	63 (17.4)	39 (16.8)	24 (18.6)	0.62
Hepatic encephalopathy	67 (18.5)	33 (14.2)	34 (26.4)	0.006
Healthcare utilization				
≥1 hospitalization in past 12 months, n (%)	109 (30.1)	54 (23.3)	55 (42.6)	0.001

Prevalence of Sarcopenia Risk

High sarcopenia risk, defined as a SARC-F score of 4 or greater, was identified in 129 patients (35.6%). The mean SARC-F score in the overall cohort was 3.2 ± 2.1. Among patients with high sarcopenia risk, the mean SARC-F score was 5.6 ± 1.2, compared with 1.9 ± 1.1 in those with low risk.

Clinical Characteristics by Sarcopenia Risk

Patients with high sarcopenia risk were significantly older than those with low risk (60.2 ± 11.3 vs 54.9 ± 12.1 years; p < 0.001). There was no significant difference in sex distribution between groups (male: 77 (59.7%) vs 144 (61.8%); p = 0.68). Patients with high sarcopenia risk had significantly higher MELD scores (14.6 ± 5.2 vs 11.2 ± 4.3; p < 0.001) and lower serum albumin levels (29.8 ± 5.1 vs 33.4 ± 4.7 g/L; p < 0.001). The prevalence of ascites was significantly higher in the high-risk group (75 (58.1%) vs 80 (34.5%); p < 0.001), as was the prevalence of hepatic encephalopathy (34 (26.4%) vs 33 (14.2%); p = 0.006). There was no significant difference in the prevalence of prior variceal bleeding between groups (24 (18.6%) vs 39 (16.8%); p = 0.62).

Healthcare Utilization

Patients with high sarcopenia risk had significantly higher rates of hospitalization in the preceding 12 months. Among those with SARC-F scores of 4 or greater, 55 patients (42.6%) had at least one hospital admission, compared with 54 patients (23.3%) in the low-risk group (p < 0.001). 

Multivariable analysis

On multivariable logistic regression analysis, three variables were independently associated with high sarcopenia risk. Older age was a significant predictor, with each additional year of age associated with a 5% increase in the odds of high sarcopenia risk (aOR: 1.05; 95% CI: 1.02-1.07; p < 0.001). Higher MELD score was also independently associated with sarcopenia risk, with each additional point conferring a 9% increase in odds (aOR: 1.09; 95% CI: 1.04-1.14; p < 0.001). The presence of ascites was associated with an 87% increase in the odds of high sarcopenia risk (aOR: 1.87; 95% CI: 1.18-2.95; p = 0.008). Model calibration assessed using the Hosmer-Lemeshow goodness-of-fit test demonstrated adequate fit (p = 0.47). The model discrimination was acceptable with a c-statistic (AUC) of 0.73, indicating moderate predictive ability. Serum albumin was significantly associated with sarcopenia risk in univariate analysis; however, it was not included in the final multivariable model due to collinearity with MELD score, which already incorporates bilirubin, creatinine, and INR as markers of liver disease severity. Sex and diabetes mellitus were not independently associated with sarcopenia risk after adjustment (Table 2).

**Table 2:** Multivariable logistic regression analysis for predictors of high sarcopenia risk OR: odds ratio; CI: confidence interval; MELD: model for end-stage liver disease

Variable	Adjusted OR	95% CI	p-value
Age	1.05	1.02-1.07	<0.001
Male sex	1.00	0.65-1.54	0.99
MELD score	1.09	1.04-1.14	<0.001
Ascites	1.87	1.18-2.95	0.008
Diabetes mellitus	1.12	0.74-1.69	0.60

## Discussion

In this cross-sectional study of 362 patients with cirrhosis at a tertiary center in Bahrain, we found that over one-third (35.6%) were at high risk of sarcopenia as identified by the SARC-F questionnaire. Sarcopenia risk was strongly associated with markers of advanced liver disease, including higher MELD scores, lower serum albumin, and the presence of ascites and hepatic encephalopathy. Furthermore, patients with high sarcopenia risk had significantly greater healthcare utilization, with nearly twice the rate of hospitalization compared with low-risk patients.

These findings are consistent with previous studies demonstrating that sarcopenia is highly prevalent in cirrhosis and is associated with adverse outcomes [3,5]. Montano-Loza et al. reported that sarcopenia, assessed by CT-based skeletal muscle index, is an independent predictor of mortality in patients with cirrhosis awaiting liver transplantation, regardless of MELD score [10]. Similarly, Tandon et al. demonstrated that frailty and muscle wasting are strongly associated with hospitalization and mortality in ambulatory patients with cirrhosis [11]. Our study extends these observations by demonstrating that a simple, patient-reported screening tool can identify high-risk patients without the need for advanced imaging, which is particularly relevant in resource-limited settings where access to CT or DEXA may be constrained.

The strong association between sarcopenia risk and MELD score observed in our study highlights the interplay between liver disease severity and muscle wasting. The pathophysiology of sarcopenia in cirrhosis is multifactorial and includes hyperammonemia-induced impairment of protein synthesis, increased autophagy, hormonal dysregulation, including reduced testosterone and growth hormone levels, systemic inflammation, and inadequate nutritional intake [6,12]. Patients with more advanced liver disease are more likely to experience these metabolic derangements, creating a vicious cycle of progressive muscle loss and hepatic deterioration.

The association between sarcopenia risk and ascites deserves particular attention. Patients with ascites often have reduced mobility due to abdominal distension and discomfort, leading to physical inactivity and accelerated muscle loss. Additionally, ascites is associated with increased energy expenditure, protein losses, and anorexia, all of which contribute to negative nitrogen balance and sarcopenia [2,13]. The finding that ascites was independently associated with sarcopenia risk after adjustment for MELD score suggests that the mechanical and metabolic consequences of ascites contribute to muscle wasting beyond what would be expected from liver disease severity alone.

The significantly higher rate of hospitalization among patients with high SARC-F scores has important implications for healthcare systems. Sarcopenia has been associated with increased susceptibility to infections, impaired wound healing, and prolonged recovery from acute illness, all of which may contribute to increased healthcare utilization [14]. Early identification of patients at risk for sarcopenia may allow targeted interventions, including nutritional optimization, physical therapy, and closer outpatient monitoring, potentially reducing the burden of hospitalization.

From a clinical perspective, routine screening for sarcopenia using SARC-F could be easily integrated into outpatient hepatology clinics [15]. The questionnaire requires no specialized equipment, can be completed in less than two minutes, and can be administered by nursing staff or completed by patients themselves in the waiting room. Patients identified as high risk could be referred for comprehensive nutritional assessment, physical therapy consultation, and consideration of pharmacological interventions such as branched-chain amino acid supplementation, which has shown promise in improving muscle mass and function in cirrhosis [16-17]. Resistance exercise programs, when feasible, have also demonstrated benefits in this population [14].

Our study has several strengths, including a relatively large sample size, consecutive patient enrollment minimizing selection bias, and the use of a validated screening tool with established cutoffs. Additionally, we assessed clinically relevant outcomes, including healthcare utilization, which has direct implications for resource allocation and patient management.

Limitations

Several limitations should be acknowledged. First, the cross-sectional design precludes causal inference, and we cannot determine whether sarcopenia risk precedes or follows the development of advanced liver disease and decompensation. Reverse causality is also possible. Advanced cirrhosis and hepatic decompensation may themselves contribute to functional impairment captured by SARC-F scores rather than sarcopenia, independently driving disease severity. As a result, the associations observed between SARC-F scores and markers of advanced liver disease should be interpreted cautiously. Longitudinal studies are needed to clarify the temporal relationship and to assess whether changes in SARC-F scores predict clinical outcomes over time. Second, it is important to note that the SARC-F questionnaire has relatively low sensitivity for detecting sarcopenia, particularly when compared with imaging-based assessments of skeletal muscle mass. As a result, the observed prevalence of 35.6% in this study may underestimate the true burden of sarcopenia among patients with cirrhosis. This potential misclassification bias likely results in underdetection of patients with reduced muscle mass but preserved functional status. Ideally, patients with high SARC-F scores would undergo confirmatory testing with CT or DEXA, though this was not performed in our study. Third, fluid retention in patients with ascites may affect patient-reported functional status, potentially leading to overestimation of sarcopenia risk in this subgroup. Fourth, this was a single-center study conducted at a tertiary referral hospital in Bahrain, and the findings may not be generalizable to other populations or healthcare settings. Finally, we did not assess dietary intake, physical activity levels, or other potentially modifiable factors that may influence sarcopenia risk.

## Conclusions

Sarcopenia risk is frequently encountered among patients with cirrhosis and appears to be associated with greater disease severity and higher use of healthcare resources. The SARC-F questionnaire offers a straightforward, low-cost, and feasible method for screening in routine clinical settings without the need for specialized equipment. While this study demonstrates significant associations between SARC-F scores and markers of disease severity, prospective studies are required to determine whether routine screening and targeted interventions translate into improved clinical outcomes.
